# Measurement of the Absolute Magnitude and Time Courses of Mitochondrial Membrane Potential in Primary and Clonal Pancreatic Beta-Cells

**DOI:** 10.1371/journal.pone.0159199

**Published:** 2016-07-12

**Authors:** Akos A. Gerencser, Shona A. Mookerjee, Martin Jastroch, Martin D. Brand

**Affiliations:** 1 Buck Institute for Research on Aging, Novato, California, United States of America; 2 Image Analyst Software, Novato, California, United States of America; 3 Touro University California College of Pharmacy, Vallejo, California, United States of America; Universidad Miguel Hernández de Elche, SPAIN

## Abstract

The aim of this study was to simplify, improve and validate quantitative measurement of the mitochondrial membrane potential (ΔψM) in pancreatic β-cells. This built on our previously introduced calculation of the absolute magnitude of ΔψM in intact cells, using time-lapse imaging of the non-quench mode fluorescence of tetramethylrhodamine methyl ester and a bis-oxonol plasma membrane potential (ΔψP) indicator. ΔψM is a central mediator of glucose-stimulated insulin secretion in pancreatic β-cells. ΔψM is at the crossroads of cellular energy production and demand, therefore precise assay of its magnitude is a valuable tool to study how these processes interplay in insulin secretion. Dispersed islet cell cultures allowed cell type-specific, single-cell observations of cell-to-cell heterogeneity of ΔψM and ΔψP. Glucose addition caused hyperpolarization of ΔψM and depolarization of ΔψP. The hyperpolarization was a monophasic step increase, even in cells where the ΔψP depolarization was biphasic. The biphasic response of ΔψP was associated with a larger hyperpolarization of ΔψM than the monophasic response. Analysis of the relationships between ΔψP and ΔψM revealed that primary dispersed β-cells responded to glucose heterogeneously, driven by variable activation of energy metabolism. Sensitivity analysis of the calibration was consistent with β-cells having substantial cell-to-cell variations in amounts of mitochondria, and this was predicted not to impair the accuracy of determinations of relative changes in ΔψM and ΔψP. Finally, we demonstrate a significant problem with using an alternative ΔψM probe, rhodamine 123. In glucose-stimulated and oligomycin-inhibited β-cells the principles of the rhodamine 123 assay were breached, resulting in misleading conclusions.

## Introduction

In healthy pancreatic β-cells insulin is secreted when elevated glucose availability increases mitochondrial energy metabolism, hyperpolarizing the mitochondrial membrane potential (ΔψM), raising the cytoplasmic ATP/ADP ratio, closing ATP-sensitive K^+^-channels (K_ATP_), depolarizing the plasma membrane potential (ΔψP), activating Ca^2+^ entry and triggering exocytosis. This is the canonical or triggering pathway of glucose-stimulated insulin secretion (GSIS). ΔψM is the major component of the proton motive force, which is an important determinant of the maximum rate of ATP synthesis or maximal ATP/ADP ratio achievable by oxidative phosphorylation. Thus, ΔψM is a key regulator of GSIS and a central intermediate between cellular energy supply and energy demand. The canonical pathway of GSIS does not explain subtleties of insulin secretion, and therefore secondary amplification or metabolic coupling factors[1] of GSIS are targets of intense research. However, most secondary coupling factors may feedback-regulate energy metabolism, and this property is currently seriously overlooked, therefore the regulation of ΔψM in GSIS requires further scrutiny. This paper describes the β-cell specific optimization and application of the absolute and unbiased ΔψM assay technology that will enable these questions to be addressed in the future.

Measurement of the magnitude of ΔψM has several important applications in β-cell and diabetes research. Firstly, semi-quantitative relationships between mitochondrial bioenergetics and insulin secretion are seemingly well established [2–8], but have been challenged [9–14]. However, only a handful of reports have performed consistent substrate titrations and compared bioenergetic and secretory parameters in a clonal insulinoma line [5], in intact rodent islets [8] and in dispersed rodent islets [15]. These studies showed that ‘energization’ of mitochondria is the best predictor of insulin secretion. Nonetheless, this idea has been largely abandoned in favor of putative downstream metabolic coupling factors [1]. However, manipulations of metabolic pathways to demonstrate such coupling factors have rarely been controlled for secondary bioenergetic effects, and if they have, they have had only limited sensitivity [13,16,17].

Secondly, comparisons of evoked changes in ΔψM using the typical semi-quantitative application of rhodamine 123 assume identical mitochondrial volume densities and baseline values of ΔψM. This makes it invalid to compare different individuals or different genetic models that may violate these assumptions. In our hands the absolute potentiometric technique allowed comparison of normal and type 2 diabetic human β-cells, leading to the identification of an imbalance between ATP turnover and substrate oxidation as a form of bioenergetic dysfunction in diabetes [18].

Thirdly, β-cells in islets [19] and in isolation [20] respond heterogeneously to rising [glucose], and this likely has physiological significance [19]. A technology that accurately measures ΔψM in single cells will allow examination of this property in various β-cell models. Data presented here indicates that cell-to-cell heterogeneity is largely driven by variable activation of energy metabolism.

To address the shortcomings of previously available ΔψM assays, particularly the confounding effects of mitochondrial volume density and differences in ΔψP between samples, we have developed a method for micro-scale single-cell analysis of ΔψM. This technology measures the absolute value of ΔψM unbiased by ΔψP. To this end the fluorescence signal of a cationic potentiometric dye (TMRM; tetramethylrhodamine methyl ester) is corrected using the fluorescence of an anionic, bis-oxonol type fluorescent ΔψP indicator (PMPI) [21]. In addition, the assay accounts for effects of cell size, residual fluorescence background, autofluorescence, mitochondrial volume density, probe binding to mitochondrial membranes, and ultrastructural differences, therefore it allows accurate comparison of different samples. Both ΔψM and ΔψP are calibrated to millivolt values from a single fluorescence time-lapse recording of single cells or populations of cells.

Fluorescence intensity changes of redistribution-type potentiometric probes reflect potentials distorted in time by their slow and potential-dependent diffusion across the plasma membrane. The calibration algorithm models this effect, and back-calculates the time course of ‘undistorted’ potentials using a set of biophysical constants that describe the movement of probes across membranes. We have previously determined and consolidated these values using electrophysiology in cortical neurons [21]. We assume that these values describing membrane permeation are constant properties of the probes, independent of the biological specimen. In contrast, the calculation of each potential requires a cell-specific rate constant (k) and a fluorescence background (f_x_) that are calculated for each fluorescence trace. Furthermore, determination of ΔψM requires knowledge of the mitochondria:cell volume fraction (V_F_) and the binding of the probe to membranes (apparent activity coefficient ratio; a_R_’, which expresses ultrastructural parameters, differences in chemical activity between the mitochondrial matrix and the cytosol, and optical dilution). The latter parameters (V_F_ and a_R_’) are cell-type specific and may be altered by disease or chronic manipulation of the specimen. They can be determined in cell populations and we suggest that this is done for each distinct specimen.

The aim of this study was to simplify, improve and validate the absolute measurement of ΔψM in pancreatic β-cells. Here we describe improvements of the established potentiometric method [21] and the optimization of the assay for single β-cells in dispersed islet cultures from rats and humans. We apply this method to demonstrate the bioenergetic heterogeneity of primary rat β-cells. Finally, we find that the semi-quantitative method of measuring ΔψM using the quench-mode rhodamine 123 signal relies on assumptions that are not met in glucose- and oligomycin-treated insulinoma cells, resulting in misleading conclusions.

## Materials and Methods

### Materials

The ΔψP indicator (PMPI; #R8042 FLIPR Membrane Potential Assay Explorer Kit) was from Molecular Devices (Sunnyvale, CA); Accutase, Geltrex, tetramethylrhodamine methyl ester (TMRM) and other fluorescence probes were from Life Technologies (Carlsbad, CA); zosuquidar was from MedKoo Biosciences (Chapel Hill, NC), and other fine chemicals were from Sigma-Aldrich (St. Louis, MO) or Santa Cruz Biotechnology (Dallas, TX), unless otherwise noted.

### Rat Islet preparation, dispersion and immunocytochemistry

Rat islets from male Wistar rats were prepared according to [22] and [23] with modifications, in accordance with IACUC standards under a protocol approved by the Buck Institute Animal Care and Use Committee (IACUC Protocol Number 10080). Briefly, the common bile duct of CO_2_-euthanized rats was injected with collagenase (type P, Roche; 1 mg/ml in Ca^2+^-free HEPES-buffered EBSS) and pancreata were digested for 20 min at 37°C. The tissue digest was triturated by a 21G needle and purified by discontinuous gradient centrifugation through Histopaque 1119, 1077 and EBSS containing HEPES (10 mM) and BSA (1 w/v %). Islets were cultured in RPMI 1640 medium supplemented with heat inactivated fetal bovine serum (10 v/v %), HEPES (10 mM), 100 units/ml penicillin and 100 μg/ml streptomycin, nonessential amino acids, pyruvate (1 mM), glutamine (2 mM) and β-mercaptoethanol (55 μM) in suspension flasks for 48 h. Islet cells were dispersed using Accutase, (1 ml, for 10 min at 37°C), triturated with a 1 ml pipette tip and plated at ~5×10^3^ cells per well in the presence of 5 v/v % Geltrex in the above medium into coverglass-bottomed, half-area 96-well microplates previously coated overnight with polyethyleneimine (1:15000 w/v %) and attached by centrifugation at 500 g for 5 min.

Experiments were performed on the 3^rd^ or 4^th^ day of dispersed culture in a non-fluorescent, custom-modified RPMI 1640 medium (Mediatech, Manassas, VA), devoid of phenol red, riboflavin and folic acid, with 5 mM NaHCO_3_ (to partially accommodate working in room air). Phosphate was lowered to enable the use of 1.5 mM CaCl_2_ and 1 mM MgCl_2_ and the medium was buffered with 20 mM TES (N-Tris-(hydroxymethyl)-methyl-2-amino-ethanesulphonic acid). Basal incubation medium contained 3 mM glucose and 2 mM glutamine. β-Cells were identified by post-hoc immunocytochemistry using guinea pig polyclonal anti-insulin primary (AR029-5R 1:5; Biogenex, San Ramon, CA) and anti-guinea pig IgG–Alexa 555 (1:500; Life Technologies) secondary antibodies.

### Human islets

Human islets (Prodo Laboratories, Irvine, CA) were cultured for 1–4 d in PIM medium (Prodo) supplemented with human serum, glutamine, and glutathione according to the supplier’s recommendations, plus 100 units/ml penicillin and 100 μg/ml streptomycin. Data were pooled from work on β-cells from four non-diabetic donors (37 yo male, BMI 26.3, Hb1Ac 5.1%; 38 yo female, BMI 30.6, HbA1c 3.5%; 33 yo male, BMI 31.4, HbA1c 5.6%; 59 yo female, BMI 31.2, HbA1c 5.3% [20]). Islet cells were dispersed in ~500 islet batches and plated at ~5×10^3^ cells per well as described above for rat β-cells.

### Insulinoma cell lines

INS-1E [24] and INS-1 832/13 cells [25] were cultured in RPMI 1640 medium containing glutamine (2 mM), pyruvate (1 mM), β-mercaptoethanol (55 μM), HEPES (10 mM), and fetal bovine serum (10 v/v %). For the microplate-based application of the potentiometric technique, an INS-1 832/13 line stably transfected with non-targeted pSilencer 4.1 CMV-puro vector was used, and 2 μg/mL puromycin was added during cell culturing and plating. Insulinoma cells were plated at 2×10^4^ cells per well on 8-well LabTek chambered coverglasses for microscopy; for the microplate assay, cells were plated at 4×10^4^ cells per well into 96-well plates (Corning 3340) 48–72 h prior to the experiment.

### Measurement of mitochondrial density and probe binding

Mitochondria:cell volume fractions (V_F_) were determined as described [21], with modifications. Briefly, cultured primary islet cells or INS-1 832/13 cells were loaded with MitoTracker Red CMXRos (75 nM) and calcein-AM (0.75 μM) for 30 min at 37°C in non-fluorescent RPMI medium, and imaged on a Zeiss LSM780 laser scanning confocal microscope using a Plan-Apochromat 63×/1.4 oil lens. 1024×1024 pixel single planes were recorded at 44 nm pixel size at 1 Airy unit pinhole at high quality. Calcein and MitoTracker Red were simultaneously excited at 488 nm and 561 nm and emissions were detected at 493–556 nm and 566–690 nm, respectively. The binding of TMRM to mitochondrial membranes at zero ΔψM (a_R_’, see Introduction) was measured using the same optical setting, as previously described [21]. For dispersed islet cultures only cells with post-hoc insulin positivity were analyzed. Recordings were analyzed using the “Mitochondria:cell volume fractionator (basic with post-hoc staining)” and “Mitochondrial membrane potential assay—measurement of the apparent activity coefficient ratio”image analysis pipelines in Image Analyst MKII (Image Analyst Software, Novato, CA).

### Fluorescence microscopic assay of ΔψP and ΔψM

Time-lapse recording was performed on a Nikon Ti-Eclipse Perfect Focus System fully motorized wide-field fluorescence microscope, equipped with a Cascade 512B camera (Photometrics, Tucson, AZ) and S-Fluor 20× air lens, a Lambda LB-LS17 Xe-arc light source, 10–3 excitation and emission filter wheels (Sutter Instruments, Novato, CA) and an MS-2000 linear encoded motorized stage (ASI; Eugene, OR), controlled by NIS Elements 4.20 (Nikon, Melville, NY). Experiments were performed at 37°C in air. To ensure that all calibrant media had the same probe concentrations, the potentiometric medium (PM) was made from a 2x Na-free stock of the non-fluorescent RPMI medium described above. TMRM (7.5 nM final), PMPI (1:200 final), tetraphenylborate (TPB; 1 μM final; to accelerate probe distribution) and zosuquidar (1 μM final; to inhibit p-glycoprotein mediated probe efflux [26]) were added to this (resulting in 2×PM) and diluted to final volume using 240 mM NaCl (resulting in PM) or alternatively KCl solutions (PMK).

Cell culture microplates were incubated in PM for 2–3 h then imaged in fresh PM. In each session, twelve view fields were cyclically imaged in six wells at 30–40 s intervals, using the following filter sets from Semrock, Rochester, NY, given as excitation–dichroic beamsplitter–emission in nm/bandwidth, for TMRM: 580/14–594–641/75 (150 ms exposure time) and for PMPI: 500/24–520–542/27 (75 ms). Mean fluorescence intensities were determined over manually drawn regions of interests (ROIs) in recordings that were background subtracted, aligned and spectrally unmixed using the “Prepare two channel time lapse for intensity analysis” standard pipeline in Image Analyst MKII. The readout was gated to be β-cell-specific using post-hoc immunocytochemistry. To this end, during image acquisition an origin was arbitrarily chosen and imaged in the plate during live-cell recording (e.g. an edge of a well). After immunostaining, this point was located using the “Live comparisons” feature of the NIS Elements software, stored coordinates were offset, and the same view fields were imaged for immunofluorescence. During image analysis, the live-cell time lapse maximum intensity projection PMPI image and the immunofluorescence images were registered, then the immunofluorescence micrograph was used as an aid for manual drawing of ROIs using the projection image as a template. ROIs were drawn sufficiently large to contain single or groups of cells even if they moved during the experiment. Previously we have shown that the way of ROI drawing (e.g. the amount of background included) does not affect the calibration [21]. To determine the magnitude of ΔψM and ΔψP at baseline and for their time course afterwards the “Membrane Potential Calibration Wizard” in Image Analyst MKII was used. Calibration constants (see Introduction) were set as given in the text. Constants not specified here were assigned previously determined values [21].

### Fluorescence microscopy of synaptopHluorin

To estimate possible optical artefacts associated with liquid handling or glucose-stimulation of β-cells, human β-cells were infected with superecliptic synaptopHluorin lentivirus [18], and pH-insensitive fluorescence was recorded using wide-field microscopy as above described with a 390/40–495–520/35 filter set.

### Fluorescence microscopy of rhodamine 123

Insulinoma cultures were incubated with 10 μg/ml rhodamine 123 for 30 min in a simple assay medium comprising of (in mM) 120 NaCl, 3.5 KCl, 1.3 CaCl_2_, 1 MgCl_2_, 0.4 KH_2_PO_4_, 5 NaHCO_3_, 1.2 Na_2_SO_4_, 20 TES, 2 glucose, 2 glutamine at pH7.4 at 37°C. Then, time lapse recording was performed in the absence of probe using a 60× Plan Apo VC NA1.4 oil immersion lens and 1.5× zoom resulting in 0.18 μm/pixel resolution using the wide-field fluorescence microcopy setup described above. To measure mitochondrial fluorescence, high pass filtering was performed using the “original X-rhod-1 mito filter.flt” in Image Analyst MKII [27].

### Plate-reader assay of ΔψP and ΔψM

The absolute magnitude of ΔψP and ΔψM in INS-1 832/13 cells were measured in a Pherastar FS (BMG) fluorescence plate reader capable of simultaneous dual emission detection, *z*-focusing and orbital averaging, equipped with a customized filter set (Semrock) comprising of a 503/572 nm dual bandpass exciter, a 444/520/590 nm multiedge excitation beamsplitter, a 562 nm emission beamsplitter, a 537/26 emission filter for PMPI and a 641/75 emission filter for TMRM. Assays were performed in a simple PM with a composition based on to the above simple assay medium at 37°C. Potentiometric calibrations were performed in Mathematica 8.0–10.2 (Wolfram Research; Champaign, IL).

### Statistical and data analysis

Data analysis was performed in Mathematica 8.0–10.2. To visualize averaged time courses, fluorescence and calibrated potential data were resampled at 30 s intervals. Statistical errors indicate SE of independent microscopy sessions unless otherwise noted. Pairwise comparisons were performed by two-tailed *t-*test. Other statistical tests are described in the text. Equilibration times were calculated by solving the differential equation of TMRM distribution across the plasma membrane, a core equation of the biophysical model of the potentiometric calibration (Eq. 18 in [21]). The equation was solved by NDSolve in Mathematica for [TMRM] in the mitochondrial matrix after expressing its cytosolic concentration as a function of ΔψM using the Nernst equation. Values for ΔψM, ΔψP and V_F_ measured in the appropriate conditions were used for the calculation. Finally, the 90% equilibration time was determined from the resultant mitochondrial matrix [TMRM] time course.

## Results

### Measurement of the mitochondria:cell volume fraction

**Fig 1:**
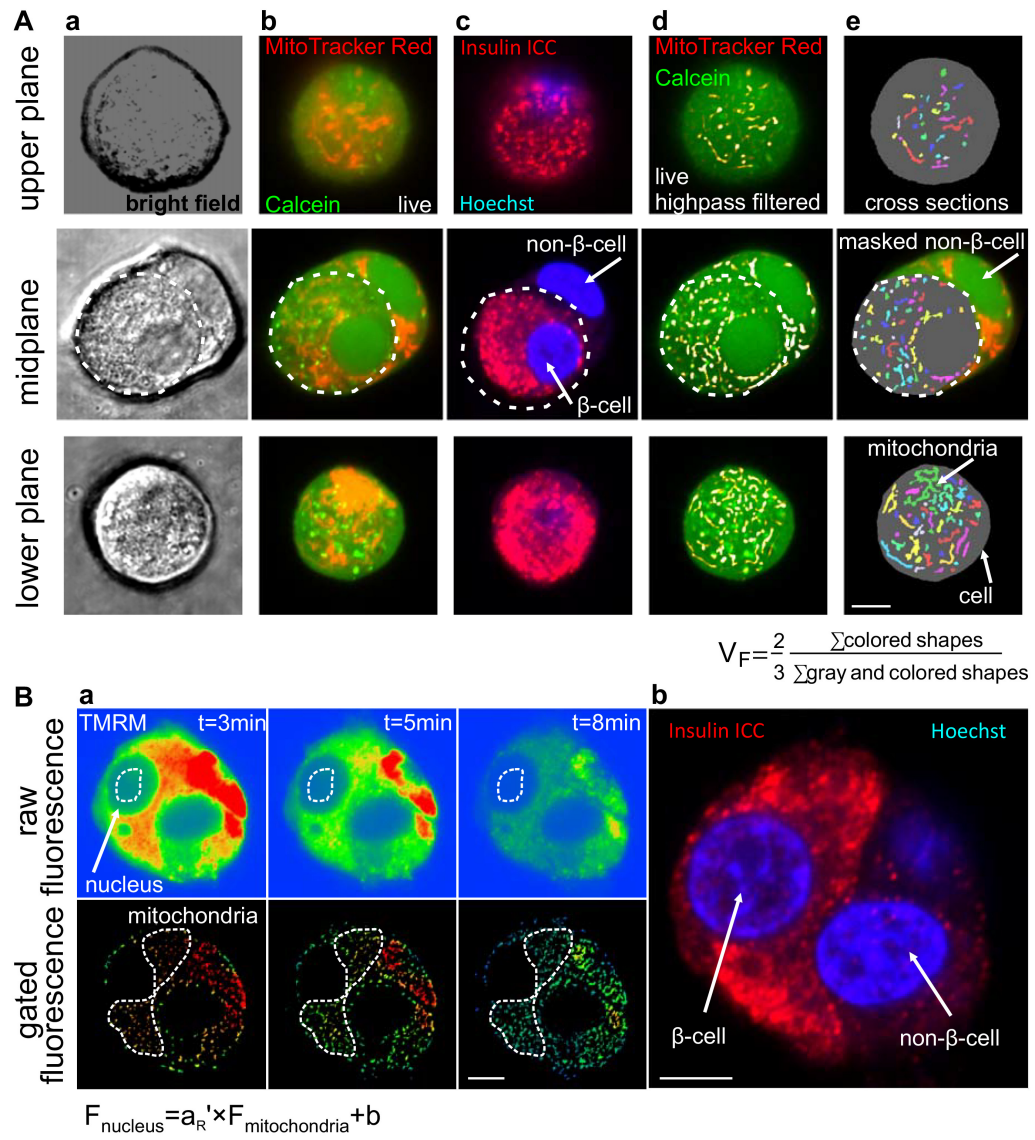
Confocal microscopic determination of geometric and affinity parameters required for the calculation of ΔψM in rat primary β-cells. (A) The mitochondria:cell volume fraction (V_F_) was calculated from confocal micrographs of live rat primary β-cells loaded with MitoTracker Red CMX and Calcein-AM (a-b). β-Cells were identified by insulin immunocytochemistry (ICC; c). MitoTracker images were high pass filtered (d) and both Calcein and MitoTracker images were binarized (e) to determine cellular and mitochondrial cross sections, respectively. Each β-cell was imaged in one of 10 serial *z*-planes to provide a balanced representation of all planes, indicated here by the three rows of images of separate cells. Cross sections of non-β cells were manually removed as indicated in the midplane images (the boundaries of the β-cell is marked by the dashed line). V_F_ was calculated by the indicated formula after summing the number of all mitochondrial and all cellular (including mitochondrial) pixels in all cells in a recording of 50–100 cells (represented by column e). (B) a_R_’ was calculated from TMRM fluorescence after depolarization of mitochondria by FCCP (1 μM), oligomycin (1 μg/ml) and antimycin A (1 μM). a_R_’ is the slope of the linear relationship between nuclear and mitochondrial fluorescence (F; as marked by the dashed line in the raw and gated fluorescence images, respectively) as TMRM fluorescence intensity decays as it leaks out of the cell, and b is background fluorescence. The identity of β-cells was determined by post-hoc immunofluorescence imaging, revisiting stored coordinates (right). Scale bars, 5 μm.

We previously developed a confocal microscopic stereological approach to determine V_F_, as an alternative to the more laborious approach of electron microscopy [21]. This was achieved by recording oversampled, high-quality images of a mitochondrial (MitoTracker Red CMX) and a whole cell stain (calcein-AM) (Fig 1Ab), followed by calculation of the ratio of the total number of mitochondrial pixels to the total number of cellular pixels counted computationally and multiplied by a stereologic correction factor of 2/3 ([21], Fig 1Ae). The stereological approach requires unbiased sampling of the cell monolayer. To avoid recording photo-damaged regions, each *x*,*y*-coordinate was imaged only once, precluding recording of *z*-stacks. Therefore a set of different cells in sparse cultures of primary β-cells was scanned while the *z*-focus was systematically cycled. To speed up recording, automation was developed. Using the Zeiss Zen Multi Time Series PLUS, *x*,*y*-coordinates of individual cells were marked under the eyepiece, then automatically imaged. Insulin immunofluorescence was recorded at the same coordinates referenced to an arbitrarily chosen origin in the sample (Fig 1Ac). During image analysis, only immunopositive cells were processed by manual ROI masking. V_F_ was 7.8±0.13% (n = 3) in Wistar rat β-cells and 6.3±0.49% in INS-1 832/13 (n = 4). For comparison, it was 7.70±0.06% in human non-diabetic subjects (n = 4 subjects) [20] and 6.94±0.08% in INS-1E cells [21]. Notably, this approach assumes that all β-cells have the same V_F_; possible deviations from this assumption are tested below.

### Measurement of TMRM binding

The apparent activity coefficient ratio a_R_’ accounts for biases in TMRM fluorescence arising from non-ΔψM mechanisms, including probe binding to mitochondrial membranes, differences in chemical activities between the cytosol and the mitochondrial matrix and the dilution of fluorescence from the matrix by the intermembrane and intracristal space. Therefore, using specimen-specific a_R_’ largely cancels the uncertainty in mitochondrial matrix volumes resolvable only by electron microscopy [21]. a_R_’ is the TMRM fluorescence intensity in the mitochondrion-free space of the nucleoplasm (Fig 1Ba top) relative to the intensity over mitochondrial profiles (Fig 1Ba bottom), at zero ΔψM, in the absence of fluorescence background or high-affinity, potential-independent binding of TMRM to cellular components. In practice, data collection was performed by recording the decay of TMRM fluorescence after discharging ΔψM. Then, a_R_’ was calculated as the slope of fluorescence intensities measured over the nuclei as a function of fluorescence intensities over mitochondria. This approach was used to cancel the effects of autofluorescence and high affinity binding that are constant in time, and therefore affect the *y*-axis intercept, but not the slope of this linear fit. This was done because autofluorescence and high affinity binding are accounted for independently, by a different term (f_x_) in the ΔψM calibration. The value of a_R_’ was 0.296±0.007 (n = 4) in Wistar rat β-cells and 0.36±0.05 in INS-1 832/13 cells. For comparison, it was 0.359±0.005 in human non-diabetic subjects (n = 4 subjects) [20].

### Potentiometric recording in rat primary β-cells

**Fig 2:**
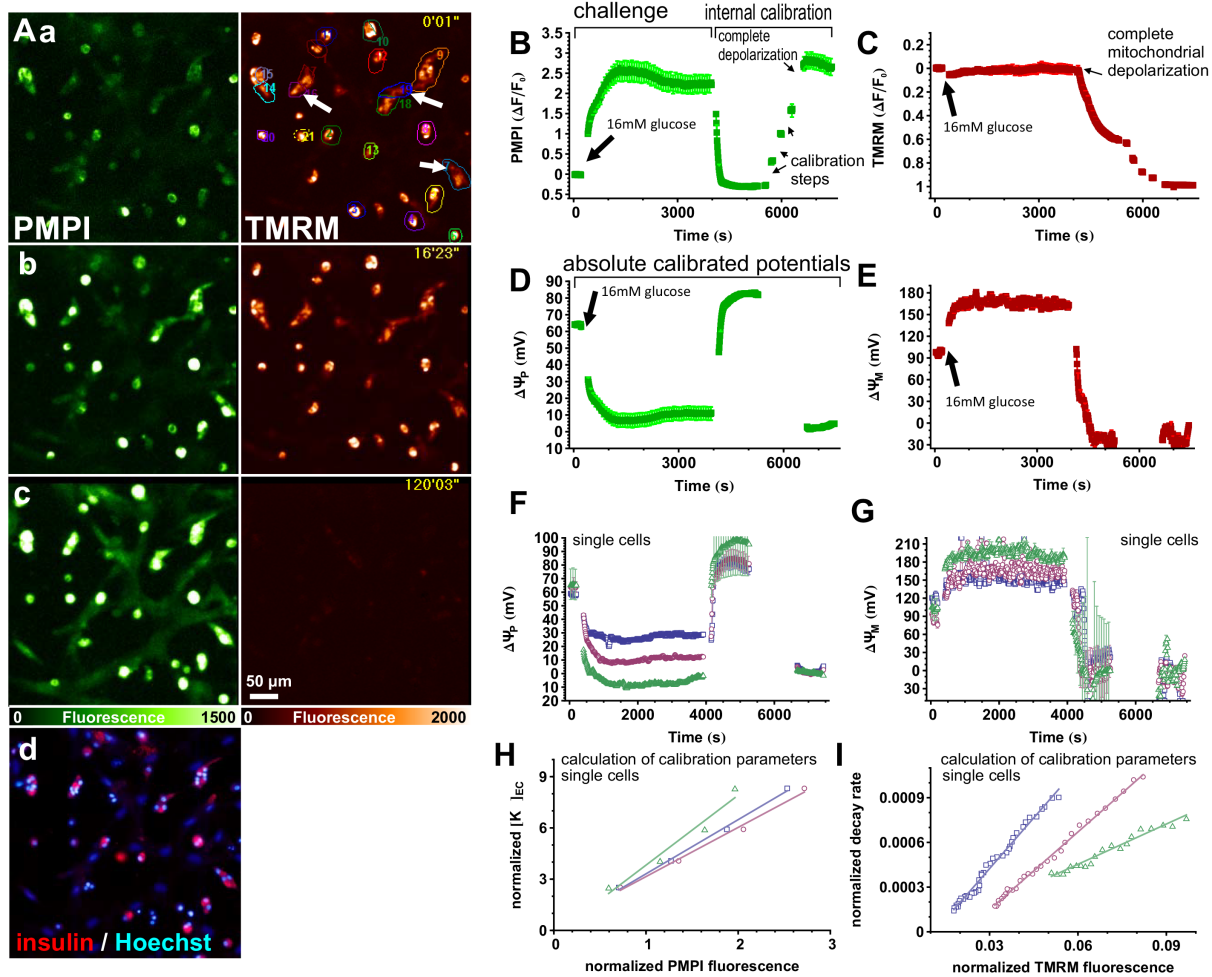
Measurement of ΔψM in rodent primary β-cells. (A) Wide-field fluorescence view field of a dispersed rat islet cell culture loaded with PMPI (left; green) and TMRM (right; red). a) 3 mM glucose baseline; b) 16 mM glucose; c) complete depolarization at the end of the experiment, corresponding to the time courses shown in B-G; d) insulin immunofluorescence (red) and Hoechst 33342 nuclear staining (blue) in the same view field. (B-C) Fluorescence intensity time courses of the potentiometric probes. (D-E) Time courses of calibrated potentials corresponding to B-C were calculated using V_F_ = 0.078 and a_R_’ = 0.296 and biophysical constants of the probes as previously published [21]. Data are mean±SE of n = 34 cells individually calibrated and pooled in five view fields in a single well, and representative of 3 independent experiments. Potentials were calibrated in single or aggregates of insulin-positive cells as indicated by regions of interests in Aa. (F-G) Calibrated potentials in 3 (groups of) cells indicated in Ab by arrows. Error bars indicate the predicted SE of the calibration calculated by propagation of the errors in determinations of calibration parameters. (H) Calculation of parameters of ΔψP calibration using linear regression on normalized fluorescence intensities and extracellular [K^+^] ([K^+^]_ec_) values during “calibration steps” shown in B. (I) Calculation of parameters of ΔψM calibration using linear regression on normalized fluorescence intensities of TMRM decay. H and I correspond to cells indicated in Aa, F and G. Normalization was performed as previously published [21].

To determine the resting ΔψM and the magnitude of its response to glucose, rat primary β-cells in dispersed islet cell cultures were preincubated in non-fluorescent, low glucose (3 mM) RPMI medium containing the potentiometric probes (PM) for ~3 h. During this time, equilibrium and steady baseline fluorescence intensity were reached. Fig 2 demonstrates the potentiometric calibration. TMRM and PMPI fluorescence were captured with wide-field fluorescence time-lapse imaging (Fig 2A). Data used for analysis were gated to insulin-containing cells (Fig 2Ad). β-Cells were challenged by elevating [glucose] to 16 mM, then an internal calibration was performed. The first step of the calibration achieves “complete mitochondrial depolarization” and sets ΔψP close to the K^+^ equilibrium (diffusion) potential (Fig 2B). To this end a mixture of oligomycin (1 μg/ml; inhibits F_1_F_o_-ATPase), valinomycin (250 nM; K^+^ ionophore; also discharges ΔψM) and antimycin A (1 μM; inhibits respiration) was applied and further supplemented by a mixture of tetrodotoxin (1 μM; inhibits voltage-gated Na^+^ channels), IAA-94 (100 μM; inhibits Cl^-^ transport), DIOA (10 μM; inhibits K^+^/Cl^-^ cotransport) and bumetanide (80 μM; inhibits K^+^/Na^+^/Cl^-^ cotransport) to mitigate cell swelling and stabilize ΔψP, as previously described [20,21]. Notably, at this step (and also in all other experiments below) [glucose] was uniformly set to 16 mM to support glycolytic ATP-production. Depolarization of ΔψM resulted in a characteristic decay profile of TMRM fluorescence that was clearly observable in a 20 min recording (Fig 2C). This decay curve encodes the baseline ΔψM into the recording, and when combined with knowledge of the end point fluorescence (below), ΔψP, V_F_ and a_R_’, allows calculation of the absolute value of ΔψM, and all other required calibration constants.

The second part of the internal calibration enables the determination of baseline ΔψP. To this end [K^+^] was incremented in several “calibration steps” up to ~80 mM by replacement of medium with PMK containing all previously added supplements (Fig 2B). Fluorescence intensities measured after each step encode [K^+^]_ic_ and the resting value of ΔψP. These steps, together with the end point fluorescence (below), allow calculation of the baseline value of ΔψP using the Goldman equation. Importantly, this requires only knowledge of external [K^+^], and no assumption of [K^+^]_ic_ is used for the calculation of ΔψP. Here we assumed that no permeabilities for ions other than K^+^ contributed to the Goldman equation (P_N_ = 0 [21]).

Finally, “complete depolarization” of both ΔψP and ΔψM was achieved by fixing cells using ~2% v/v paraformaldehyde containing PMK supplemented with the Na-ionophore gramicidin (10 μg/ml; Fig 2B). Both ΔψM and ΔψP calibrations (above) require these end-point fluorescence intensities.

### Calibration of potentials in single cells with quality control

The calculation of potentials from PMPI and TMRM fluorescence intensity time courses was performed using the “Membrane Potential Calibration Wizard” of Image Analyst MKII. Fig 2D and 2E show the average response of 34 cells in a representative experiment. Calibrating single cell data allowed for quality control (QC) for healthy cells and analysis of the responses of individual cells. QC was partly based on “normal behavior” of cells throughout the calibration procedure. The software calculates absolute values of potentials in mV using equations given in [21]. The calibration parameters for these equations are determined for each cell automatically by two linear regressions, one to obtain parameters required to calculate baseline ΔψP (Fig 2H) and one to calculate baseline ΔψM (Fig 2I). The membrane potential-dependent distribution of the probes dictates linear relationships between these graphed pairs of normalized values (for details of the normalizations see [21]). Importantly, the “normal behavior” of cells is defined by the underlying biophysical predictions that dictate these linear relationships. An individual cell may deviate from this, for example, if it loses plasma membrane integrity during calibration, or if the fluorescence signal is compromised by cell motility, wash-off or debris. These cells were excluded by QC, and we used a linear fit criterion of r^2^>0.2 to pass QC.

Another practical QC criterion is the precision of the calibration that can be evaluated by the standard error associated with baseline potentials. Standard errors were calculated for each data point in a single-cell trace (Fig 2F and 2G), using propagation of errors originating from noise in intensity measurements, the precision of linear fitting, and the SE of other parameters of the calibration, such as V_F_. Error estimation is implemented in the “Membrane Potential Calibration Wizard” of Image Analyst MKII using standard error propagation through published calibration equations [21]. As standard QC criterion we excluded traces with baseline SE of ΔψP or ΔψM >25 mV. We also excluded traces in which baseline ΔψP was more depolarized than -40 mV (in 3 mM glucose).

Notably, for the calibration of ΔψP one of the required parameters is the rate constant (k_P_), which expresses the rate of PMPI uptake at zero potential. However, since the changes in PMPI intensity (and ΔψP) were relatively slow, disequilibrium of the probe was not expected to substantially distort the readout, and the actual value of k_P_ did not noticeably affect the calibration. Therefore we used the previously-determined value of 0.38s^-1^ [21]. This approach is dubbed the “Complete with known k_P_ (K-steps)” ΔψP calibration method. For calculation of ΔψM the “Complete” calibration algorithm was used. In contrast, the rate constant of TMRM distribution through the plasma membrane at zero ΔψP (k_T_) is calculated from each fluorescence trace by deriving it from the regression parameters in Fig 2I. The value of k_T_ was 0.015±0.002 s^-1^ in rat primary β-cells (n = 6 experiments) and 0.015±0.001 s^-1^ human primary β-cells (n = 7 individuals, healthy or diabetic, from [20]). This k_T_ value translates to 10 min time to 90% equilibration of TMRM across the plasma membrane when mitochondria are completely depolarized from potentials shown in Fig 2D and 2E before complete mitochondrial depolarization, and we used a 20 min time-lapse recording to capture this. This k_T_ value also predicts that 90% and 99% equilibration takes 34 and 67 min, respectively when cells are initially incubated with TMRM in the presence of TPB to speed up TMRM distribution at the resting potentials given below (see [28] for TMRM equilibration times in the absence of TPB).

### Optimization of the assay for use with β-cells in primary cultures of dispersed islet cells

The temporal resolution required for complete, absolute calibration of ΔψM is dictated by the speed of decay of TMRM fluorescence after complete mitochondrial depolarization. This decay is relatively slow (~ 10 min to 90% equilibration, see above) in primary β-cells because of their small surface:volume ratio. The resolution of the TMRM fluorescence decay is a requirement for the “Complete” calibration to calculate k_T_ and then the baseline ΔψM. The quality of the linear regression derived from this decay recording shown in Fig 2I establishes a practical frame rate requirement, but at slower cycle rates calibration can be performed if k_T_ is already known (Gerencser, unpublished). The length of the decay allowed parallel recording of 12 view fields in six wells of a 96-well microplate at a rate of ~35 s/cycle with our microscope configuration. Notably, k_T_ allows conversion of disequilibrium TMRM fluorescence (including during baseline) into potentials.

The composition of the cocktail used for “complete mitochondrial depolarization” was simplified and optimized for β-cells. Valinomycin provides sufficient uncoupling of oxidative phosphorylation, so the protonophoric uncoupler FCCP previously used in this cocktail [21] was omitted. This resulted in a more stable and better polarized ΔψP during the calibration, possibly because addition of FCCP generated a significant contribution of proton diffusion potential to ΔψP or facilitated K^+^/H^+^ exchange and loss of [K^+^]_ic_. Calibrated baseline values were not different when different valinomycin concentrations (100–1000 nM) were used, (data not shown), suggesting that K^+^ equilibrium (diffusion) potential was reached. Notably, in β-cells K_ATP_ channels may contribute substantially to plasma membrane K^+^ permeability in this deenergized condition.

Next, the composition of the cocktail used for complete depolarization was simplified and optimized. Use of the original complex cocktail [21] often caused a loss of PMPI fluorescence in primary β-cells. Therefore we switched to using paraformaledehyde (PFA) to stop active ion pumping and discharge potentials. An 8% v/v solution of PFA was mixed with 2×PM and 2 M KCl in appropriate ratios to provide 1× probe concentrations and ~125 mM KCl. This solution was then used to replace half of the PM over the cells. PFA did not interfere with PMPI fluorescence or its quencher as judged from the stability of the background fluorescence and similar dynamic range of PMPI fluorescence as with the original cocktail. Using PFA is a substantial simplification compared to our original method [21]. Importantly, it facilitates protocols that include subsequent immunostaining for post-hoc cell type identification.

We noticed that both TMRM and PMPI could be significantly depleted (by 10–20%) by handling of PM in polypropylene tubes and reservoir plates. The depletion can be detected by measuring background fluorescence (before performing background subtraction). Therefore all vessels were pre-incubated and rinsed with PM, or alternatively, a correction for depletion of probes was applied during calibration, based on changes in background fluorescence intensity using the “Expert overrides” tab in the “Membrane Potential Calibration Wizard”.

### Optimization of the length of culturing of human β-cells

**Fig 3:**
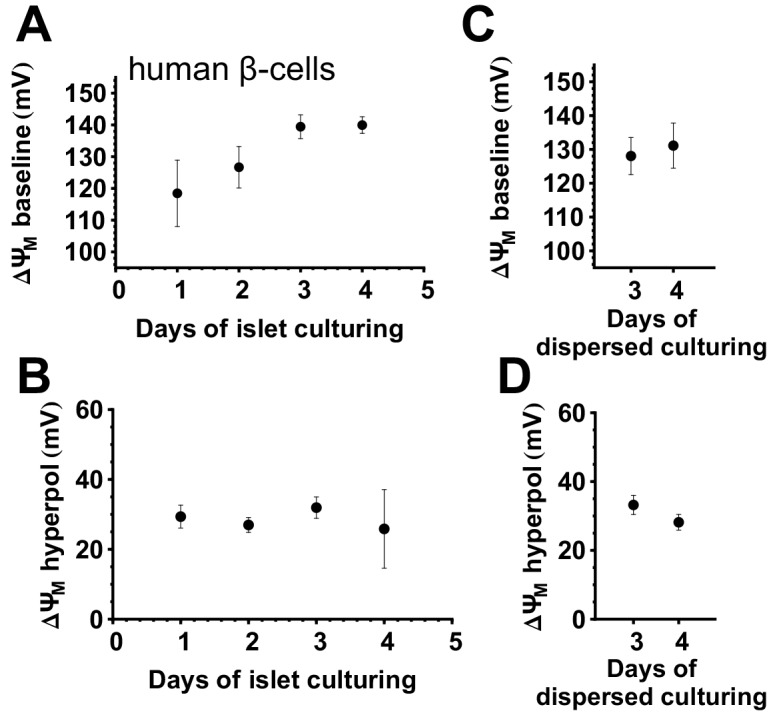
Stability of ΔψM during culturing of human primary β-cells in whole islet and dispersed cell cultures. (A) ΔψM in 3 mM glucose (rich medium) as a function of days spent in whole-islet suspension culture before dispersion. ΔψM was measured 3–4 days after dispersing islet cells. (B) ΔψM hyperpolarization evoked by 16 mM glucose corresponding to (A). (C) ΔψM in 3 mM glucose (rich medium) as a function of days spent in dispersed islet cell culture after an arbitrary length of whole islet culturing. (D) ΔψM hyperpolarization evoked by 16 mM glucose corresponding to (C).

The dependence of the basal and glucose-stimulated values of ΔψM on the duration of islet and cell culturing was studied in human β-cells in primary dispersed islet cultures from non-diabetic organ donors. Isolation stress and adaptation to culturing conditions may affect the physiology of primary β-cells. Therefore we looked at how basal ΔψM at 3 mM glucose in the presence of 2 mM glutamine and other amino acids present in the RMPI 1640 medium, and its response to elevation of glucose to 16 mM, changed with days in culture. Basal ΔψM (Fig 3A), but not the extent of hyperpolarization evoked by 16 mM glucose (Fig 3B) increased with time of islet culturing (day 0 was defined as the day islets were received after overnight shipping from Prodo Laboratories). Resting ΔψM was dependent on the whole islet suspension culturing time, but not on dispersed islet culturing time (Fig 3C and 3D), and was less correlated to the time of organ procurement or islet purification (not shown). Notably, in our recent study using the same specimens [20] we drew conclusions based on data relative to baseline. Rat islets were dispersed after 2 d in culture in this study.

### Membrane potentials in rat primary β-cells and their response to glucose

**Fig 4:**
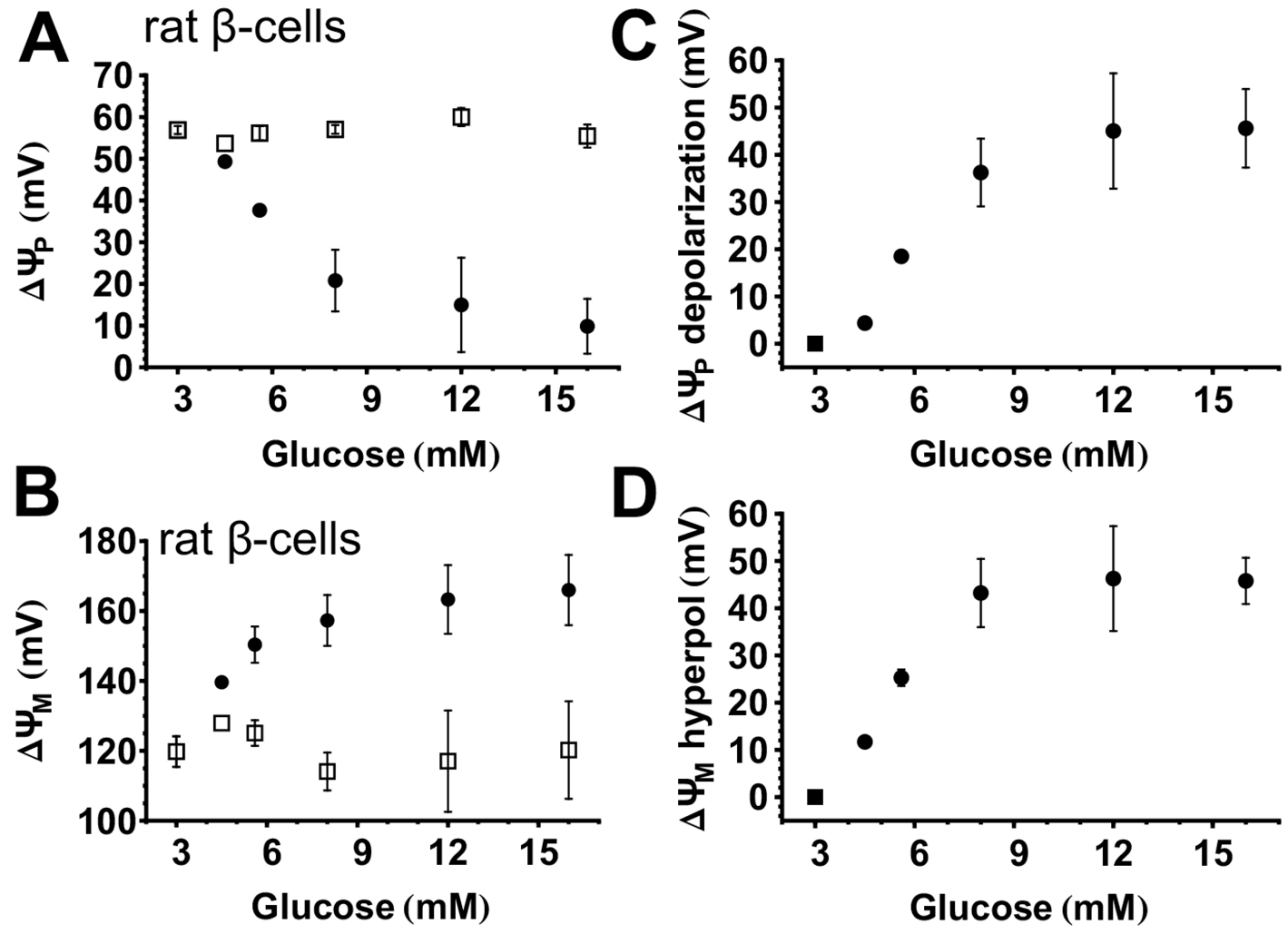
Concentration-dependent hyperpolarization of ΔψM and ΔψP by glucose in rat primary β-cells. (A-B) Absolute magnitudes of ΔψP and ΔψM. For each [glucose], first the baseline (open squares) was recorded in the presence of 3 mM glucose in a rich medium then [glucose] was elevated to the indicated value (closed circles). (C-D) Depolarization of ΔψP and hyperpolarization of ΔψM relative to baseline. The square marks the zero value, while closed circles mark the relative change from the baseline. Data are mean±SE of n = 3 experiments conducted similarly to the one shown in Fig 2. The response to glucose was determined as the mean of potentials measured at 30–60 min after addition of glucose.

Using the paradigm described above in Fig 2 the magnitudes of ΔψP and ΔψM and their responses to glucose were studied in rat primary β-cells. After recording a ~15 min baseline, [glucose] was elevated to 4–16 mM without altering probe concentrations in parallel wells of the microplate (Fig 4). This was followed by the internal calibration shown in Fig 2, and potentials were then calculated only in insulin-positive cells. The mean resting ΔψM at 3 mM glucose in the presence of amino acids including glutamine was -120±4 mV (n = 3 experiments) and this hyperpolarized to -166±10 mV (n = 3 experiments) upon exposure to 16 mM glucose. The potentiometric calibration was performed independently, and internally for each glucose concentration. All recordings were started at identical baseline conditions, so that even though treatments following baseline were different, the internal calibration performed at the end of the time lapse is expected to yield identical baseline potentials. Open symbols in Fig 4A and 4B indicate that there was no significant difference between baseline potentials. Relative responses (calculated as the difference between potentials at baseline and at 30–60 min) are shown in Fig 4C and 4D.

### Glucose-evoked ΔψM is always monophasic in rat β-cells

**Fig 5:**
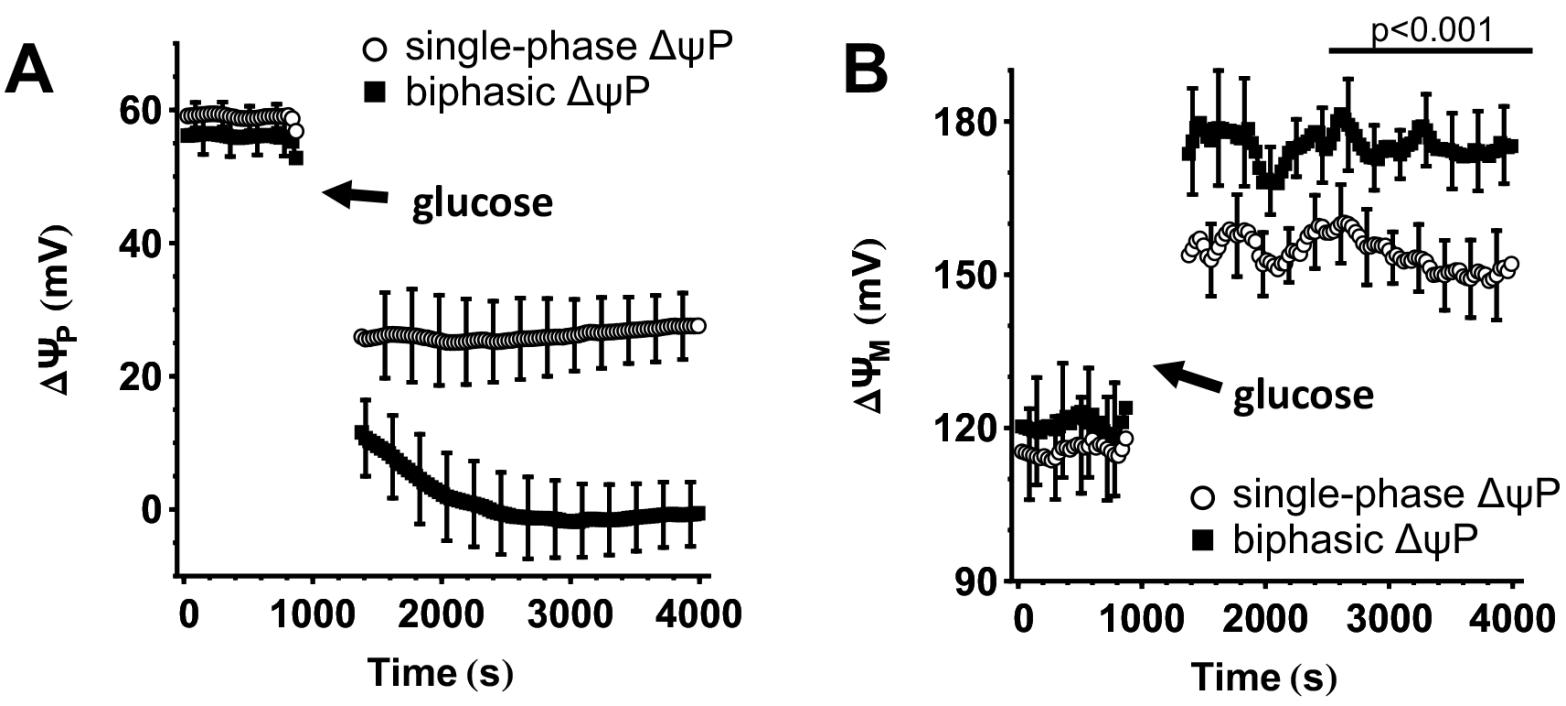
Biphasic glucose-induced ΔψP depolarization is linked to stronger, but monophasic ΔψM hyperpolarization. (A) ΔψP in glucose-stimulated rat β-cells. β-Cells were categorized into two groups based on ΔψP with monophasic (open symbols) or biphasic (closed symbols) response to 12–16 mM glucose. (B) ΔψM in β-cells categorized based on ΔψP in (A). Data are mean±SE of n = 4 experiments where individual categorized cells within each experiment were expressed as a single mean value. The p-value compares ΔψM (averaged in the interval of 30–60 min after glucose stimulation) between the two groups using paired *t*-test for experimental replicates.

Performing the potentiometric calibration on single cells allows the classification of β-cells with differing responses. Representative single-cell recordings (Fig 2F) indicated that glucose-stimulated ΔψP-depolarization in some cells reaches a plateau in ~5 min (defined as monophasic), while in others, after the initial depolarization, ΔψP further depolarizes, reaching a plateau in ~30 min (defined as biphasic). We observed oscillations in only a few rat β-cells. To investigate the bioenergetic basis for the biphasic ΔψP response to 16 mM glucose we categorized β-cells into monophasic and biphasic responders (Fig 5A). Biphasic response was defined by a ΔψP at 30 min that was at least 10% more depolarized than in the first 5 min. Remarkably, biphasic responders exhibited larger mitochondrial hyperpolarization than monophasic responders from the beginning of the glucose stimulation, but the response of ΔψM to glucose was monophasic in both cases (Fig 5B).

### Heterogeneity of the response of individual rat β-cells to glucose

**Fig 6:**
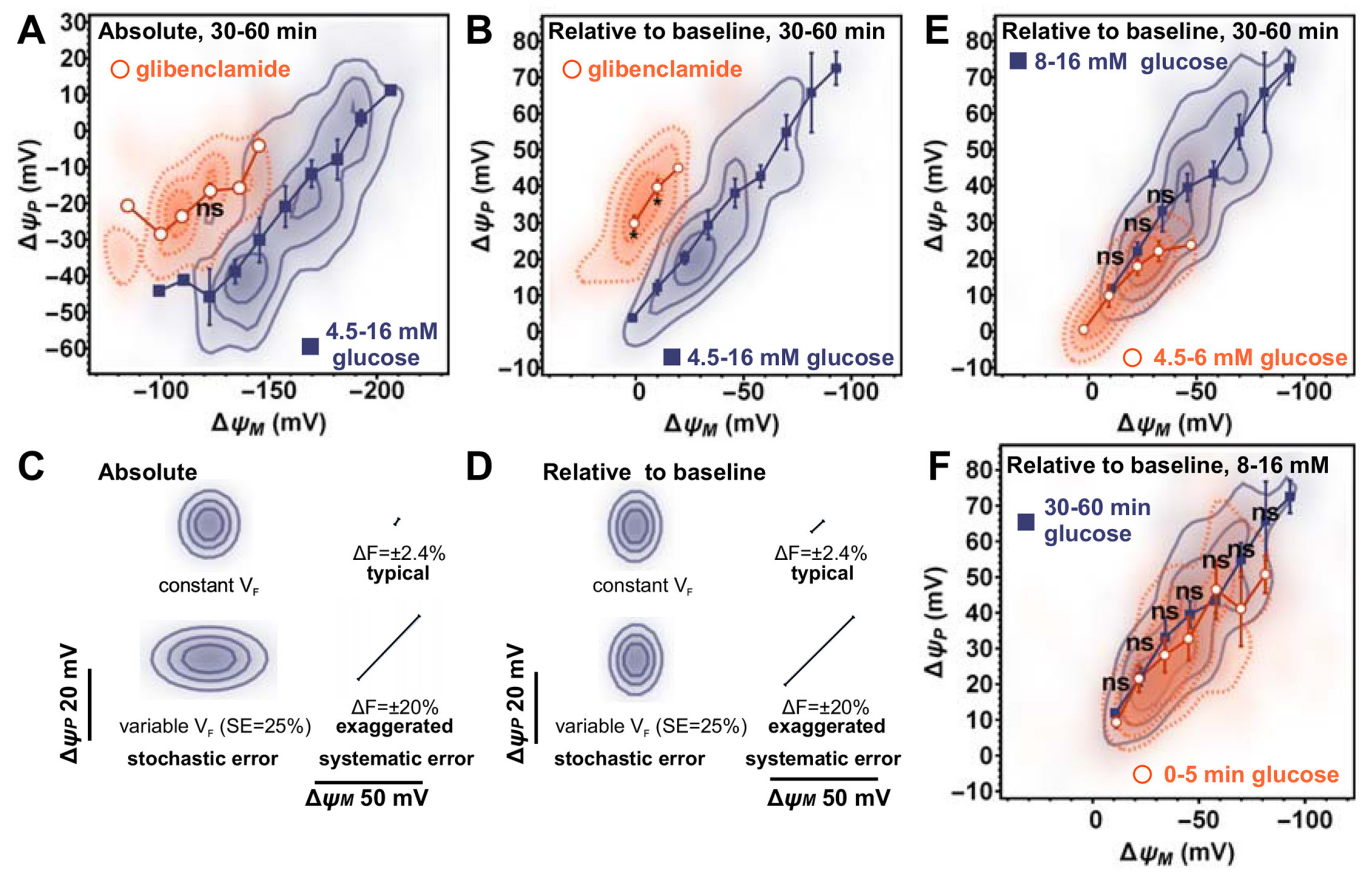
Heterogeneous response of individual rat β-cells to glucose. (A) ΔψP as a function of ΔψM in the presence of 4.5–16 mM glucose (solid isolines and closed squares) or 50 μM glibenclamide (dotted isolines and open circles). Two-dimensional histograms show the distribution of absolute potentials measured 30–60 min after secretagogue addition. Histograms were compiled from 364 and 55 cells for glucose and glibenclamide, respectively, pooled from 3 experiments (spanning the range of 4.5–16 mM [glucose] in each experiment using different wells, corresponding to data in Fig 4). Symbols indicate mean±SE of potentials calculated by binning cells along the ΔψM axis (n = 3 experiments). (B) ΔψP depolarization relative to baseline as a function of ΔψM hyperpolarization relative to baseline evoked by glucose or glibenclamide calculated from data in (A). (C-D) Predicted uncertainty of single-cell potential calibration when the absolute magnitude of stimulation-evoked potentials (C; corresponding to A) or the relative changes to baseline (D; corresponding to B,E,F) are calculated. The distributions marked by isolines visualize stochastic uncertainty originating from detection error assuming constant (top distribution) or cell-to-cell variable V_F_ (bottom distribution). Straight lines indicate the direction and magnitude of the predicted SE of a stimulation-evoked transient change in the efficiency of collecting single-cell fluorescence of the indicated magnitude. Notably the standard error of the stimulation-evoked total fluorescence change measured using the pH-insensitive fluorescence of superecliptic synaptopHluorin corresponds to the top line. (E) Relationship between relative potential changes evoked by low glucose (4.5–6 mM; dotted isolines and open circles; 79 cells) and high glucose stimulation (8–16 mM; solid isolines and closed squares; 285 cells). (F) Relationship between relative potential changes evoked by high glucose (8–16 mM; 285 cells) in the first phase of stimulation (0–5 min; dotted isolines and open circles) and in the plateau phase of the stimulation (30–60 min solid isolines and closed squares). (A-B,E-F) For each bin, values of ΔψP in the two groups were compared by *t*-test performed on the experimental repeats (*p<0.05; ns, not significant. p is not indicated for bins where data were insufficient).

The heterogeneous response of the β-cell population to glucose is likely to play a role in tuning the sensitivity of the islet [29,30]. We generally observed that individual β-cells had widely different absolute responses of ΔψP and ΔψM to a single [glucose] in our recordings in rat (Figs 2 and 5) and in human β-cells [20]. In contrast to the stimulation by single concentrations of glucose, Fig 6A visualizes the distribution of ΔψP and ΔψM over a range of [glucose] (4.5–16 mM; blue squares; solid isolines) and a single glibenclamide concentration (50 μM; red circles, dotted isolines) in rat β-cells during the plateau of ΔψP depolarization. We observed a continuous spread of the distribution of glucose-stimulated ΔψM in individual cells as opposed to discrete responses corresponding to each [glucose]. The spread of the potentials is a hallmark of heterogeneous single-cell response. We found an essentially similar relationship of ΔψP and ΔψM in human β-cells treated with a single 16 mM glucose concentration [20].

An important question is the degree to which any detection error and unaccounted-for cell-to-cell biological variability, such as potentially differing V_F_, contribute to the observed variability. Calculation of relative change for each cell may cancel effects of factors that differ between individual cells. To assess this, besides plotting the absolute, plateau potentials (Fig 6A), the relative-to-baseline, glucose- and glibenclamide-evoked changes are shown in Fig 6B. Next, we estimated the error of the potentiometric calibration for each individual cell as described above. In this data set, for the absolute plateau of ΔψM, the estimated SE was 6.2±1.5 mV for single cells, and for the determination relative to baseline, it was 4.7±0.6 mV (n = 4 experiments; cells with predicted error >25 mV were rejected by QC). To visualize the effect of the predicted inaccuracy of the calibration, 2-dimensional Gaussian distributions with SDs equal to the estimated SE along the ΔψM and ΔψP axes were created and plotted with the same algorithm and scaling as for Fig 6A and 6B in Fig 6C and 6D. These distributions show how much of the variation in the measured single-cell responses is likely due to effects of stochastic detection errors (top distributions in Fig 6D and 6E). Importantly, the relatively small variation caused by errors in the calibration highlight that the heterogeneity we observed is due largely to biological variation and not to measurement artefacts.

A possible biological variation between individual cells may be variable amounts of mitochondria, but the potentiometric calibration assumed an identical V_F_ value. Therefore we modeled the error potentially caused by a ±25% variation of V_F_ between cells (bottom distributions in Fig 6D and 6E). Remarkably, the estimated error in the relative-to-baseline ΔψM calculation was insensitive to variations of V_F_, but such variation can introduce a substantial uncertainty into the calculated absolute value of ΔψM. Thus, effects of varying amounts of mitochondria per cell, and to a smaller extent, effects of recording noise are consistent with the observed wider spread of the absolute potentials than the relative potentials (Fig 6A and 6B). Importantly, variations in V_F_ and measurement noise cannot explain the observed extent of variability of glucose stimulation. The tighter distribution of ΔψM in glibenclamide-stimulated β-cells (Fig 6B) further supports that the observed variability is not generic to the assay, but is specific for the biology of glucose stimulation.

Next, we exclude possibility that systematic errors may inject a pattern into the distributions shown in Fig 6. The measurements of ΔψP and ΔψM are not independent. The goal of the potentiometric technique is to cancel the effects of ΔψP on TMRM fluorescence intensities, but this correction may be insufficient due to measurement errors. The analysis of errors discussed above showed that stochastic, independent errors in calibration of ΔψP cannot explain the observed relationship of ΔψP and ΔψM. Furthermore, published sensitivity analysis [21] indicates that erroneous calibration of ΔψP leads to systematic errors with the same sign in ΔψP and ΔψM, thus would cause a spread of distribution perpendicular to that observed in Fig 6.

A considerable source of systematic error is the possibility of inaccurate collection of the total fluorescence intensity from each single cell. The measurement may be confounded by cell motility, shape changes, changes of focus or illumination intensities. This is a critical source of error, because an artefactual, concomitant rise in both TMRM and PMPI fluorescence would mimic ΔψP depolarization and ΔψM hyperpolarization, and could explain the observed relationship between ΔψP and ΔψM. Such changes are possible during liquid handling, e.g. when cultures are stimulated by glucose, or during the calibration procedure. The effects of simultaneous, proportional TMRM and PMPI fluorescence changes were modeled by manipulating experimental data. Modeling indicated that a single fluorescence change artefact cannot cause an association between ΔψP and ΔψM in both absolute and relative plots (not shown). Absolute calibrated potentials would be unaffected by any change in fluorescence collection before the time period in which potentials are determined (e.g. 30–60 min after stimulation to plot data in Fig 6A), but would be affected if the change happened during the “complete mitochondrial depolarization”. Artefacts occurring later in the calibration paradigm contribute little to the error that mimics an inverted relationship of the two potentials (perpendicular to that observed in Fig 6A). In contrast, the relative-to-baseline calibration can be biased by a change in fluorescence collection between baseline and the measurement period, but is little affected if such a change happens afterwards. Altogether, the marked relationship we observe between glucose-stimulated ΔψP and ΔψM in both absolute and relative-to-baseline plots excludes the possibility that a single artefactual change in efficiency of fluorescence detection causes the observed phenomenon.

It remains a theoretical possibility that if the efficiency of fluorescence collection changes upon glucose addition, and this change is reversed upon “complete mitochondrial depolarization” then this may cause the diagonal spread of potentials both in Fig 6A and 6B. To address this possibility, in separate experiments using a fluorophore with constant brightness (the pH-insensitive, UV-excited peak of superecliptic synaptopHluorin) the effect of glucose stimulation on fluorescence intensities was measured in human primary β-cells. The SD of glucose-evoked change was 2.4%. We modeled the effect of a ±2.4% (and ±20% for comparison) simultaneous change in fluorescence emission of TMRM and PMPI for the duration of glucose addition on ΔψP and ΔψM (Fig 6C and 6D, diagonal lines). None of these could explain the spread of the distribution of glucose-evoked potentials. Altogether, the sensitivity analysis described above provides solid support for the proposition that the visualized heterogeneity of glucose-evoked ΔψP and ΔψM validly reflects biological changes in membrane potentials in single cells.

### Constant relationship between ΔψP and ΔψM during glucose stimulation

The simultaneous assay of ΔψP and ΔψM in single cells allows mapping of their relationship, and the sensitivity analysis described above has validated this approach. The fact that ΔψP and ΔψM are linked under glucose stimulation highlights the operation of the canonical pathway of GSIS, where ΔψM hyperpolarization raises cytoplasmic ATP/ADP ratios, closing K_ATP_ and depolarizing ΔψP. This linkage is suspended by artificially closing K_ATP_ with glibenclamide, resulting in a smaller variation of measured potentials than in glucose-stimulated β-cells (Fig 6B).

Previously we used this technology to compare human β-cells from type 2-diabetic and non-diabetic individuals, and found no difference in the relationship between ΔψP and ΔψM, indicating intact signaling between ΔψM and K_ATP_ in diabetes [20]. Because of the heterogeneous response of ΔψM to glucose, it was possible to observe this relationship using a single [glucose]. In contrast, in Fig 6A and 6B, data from rat β-cells were pooled from a series of [glucose] shown in Fig 4 in order to collect a sufficient number of cells to plot the distribution. This recapitulated data from non-diabetic human β-cells [20]. Fig 6E indicates that stimulations by 4–6 mM and 8–16 mM glucose follow the same relationship between ΔψP and ΔψM, differing only in the extent of response. Furthermore, Fig 6F compares the distribution of potentials in the first 5 min to those in the 30–60 min window of the glucose stimulation. ΔψP at identical ΔψM was equal between the first and second phases of stimulation for most of the span of possible ΔψM values, but the first phase had a tendency towards a smaller ΔψP depolarization at larger ΔψM hyperpolarization. This tendency appeared as a significant difference in Fig 5.

Altogether, these findings are consistent with the proposition that the operation of the canonical pathway of GSIS is depicted by this assay. The variable extent of ΔψP depolarization by different [glucose] is sufficiently explained by glucose-evoked changes in ΔψM, as different cells with the same ΔψM hyperpolarization have the same ΔψP depolarization. However, the above tendency and the observed behavior of biphasic ΔψP responders may indicate the operation of secondary coupling mechanisms in cells with highly hyperpolarized ΔψM in the second phase of the stimulation.

### Experiments using rhodamine 123 in quench mode give misleading results in oligomycin-treated INS-1 832/13 cells

**Fig 7:**
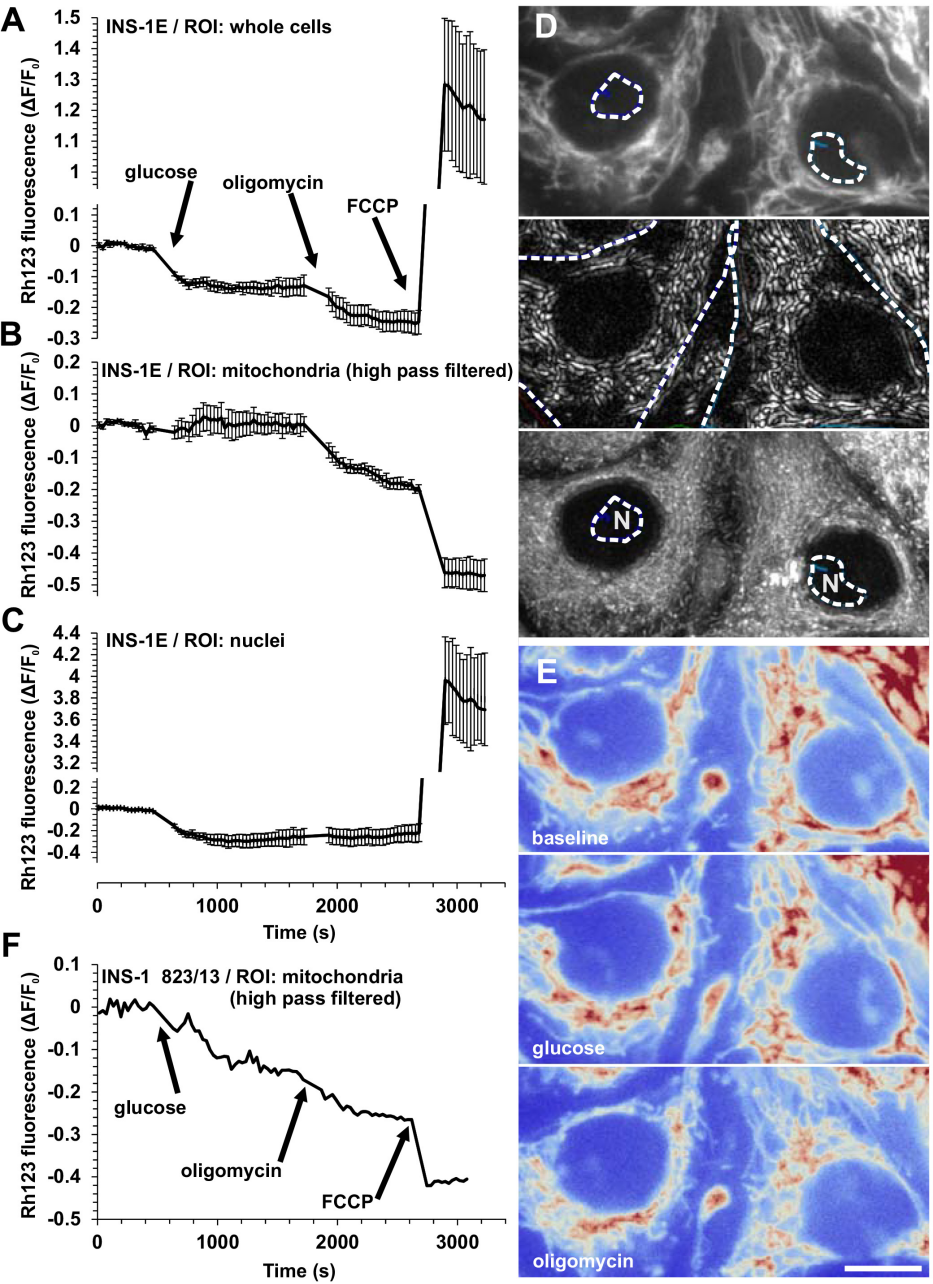
Assaying the effect of oligomycin on ΔψM using rhodamine 123 in quench mode in insulinoma cells results in internally inconsistent data. (A) Whole cell fluorescence, (B) mitochondrial fluorescence obtained by high pass filtering and (C) fluorescence over the nuclei in rhodamine 123-loaded INS-1E cells following the additions of glucose (30 mM), oligomycin (2 μg/ml) and FCCP (1 μM). Data are mean±SE of n = 3 experiments. Note that the oligomycin-evoked drop in quench-mode whole-cell fluorescence (A) originates from the mitochondria (B) and not from the cytosol (approximated by rhodamine 123 fluorescence in the nucleosol) (C). (D) Representative images of INS-1E cells. From top to bottom: raw fluorescence, high pass filtered fluorescence to selectively transmit mitochondrial signal, and the temporal maximum intensity projection of the latter image series indicating that no mitochondria moved into the ROIs (dashed outlines) used to measure nuclear fluorescence (N). (E) Raw fluorescence, corresponding from top to bottom to baseline, glucose and oligomycin treatments. The red color indicates high intensity. (D-E) Scale Bar, 10 μm. (F) Mitochondrial fluorescence obtained by high pass filtering in INS-1 832/13 cells in the same condition as above. Representative trace of three experiments.

The quench mode potentiometric measurement of rhodamine 123 has been widely used for qualitative and semi-quantitative determination of relative changes in ΔψM in β-cell research [5,6,31,32]. This approach offers a largely ΔψP-independent determination of acute changes in ΔψM, such as during glucose stimulation and following oligomycin treatment of insulinoma cells (Fig 7A). The principle of the quench-mode ΔψM assay is that the fluorescence originating from mitochondria is constant, and detected total fluorescence changes reflect alterations in the probe concentration in the cytosol, which is in Nernstian equilibrium with the probe concentration in the mitochondrial matrix. This principle allows a decrease in total cellular fluorescence to be interpreted as an increase in ΔψM, while an increase in fluorescence indicates a decrease in ΔψM. In Fig 7A, therefore, the whole cell fluorescence traces suggest that glucose hyperpolarizes ΔψM, which is then further hyperpolarized by oligomycin and finally depolarized by the uncoupler FCCP. Importantly, this interpretation is absolutely dependent on the assumption that the quench-mode mitochondrial fluorescence, governed by the quench limit, is constant. Even a small change in quench-mode mitochondrial fluorescence may invalidate this interpretation, because the total fluorescence originating from mitochondria is at least an order of magnitude more intense than the fluorescence originating from the cytosol.

**Fig 8:**
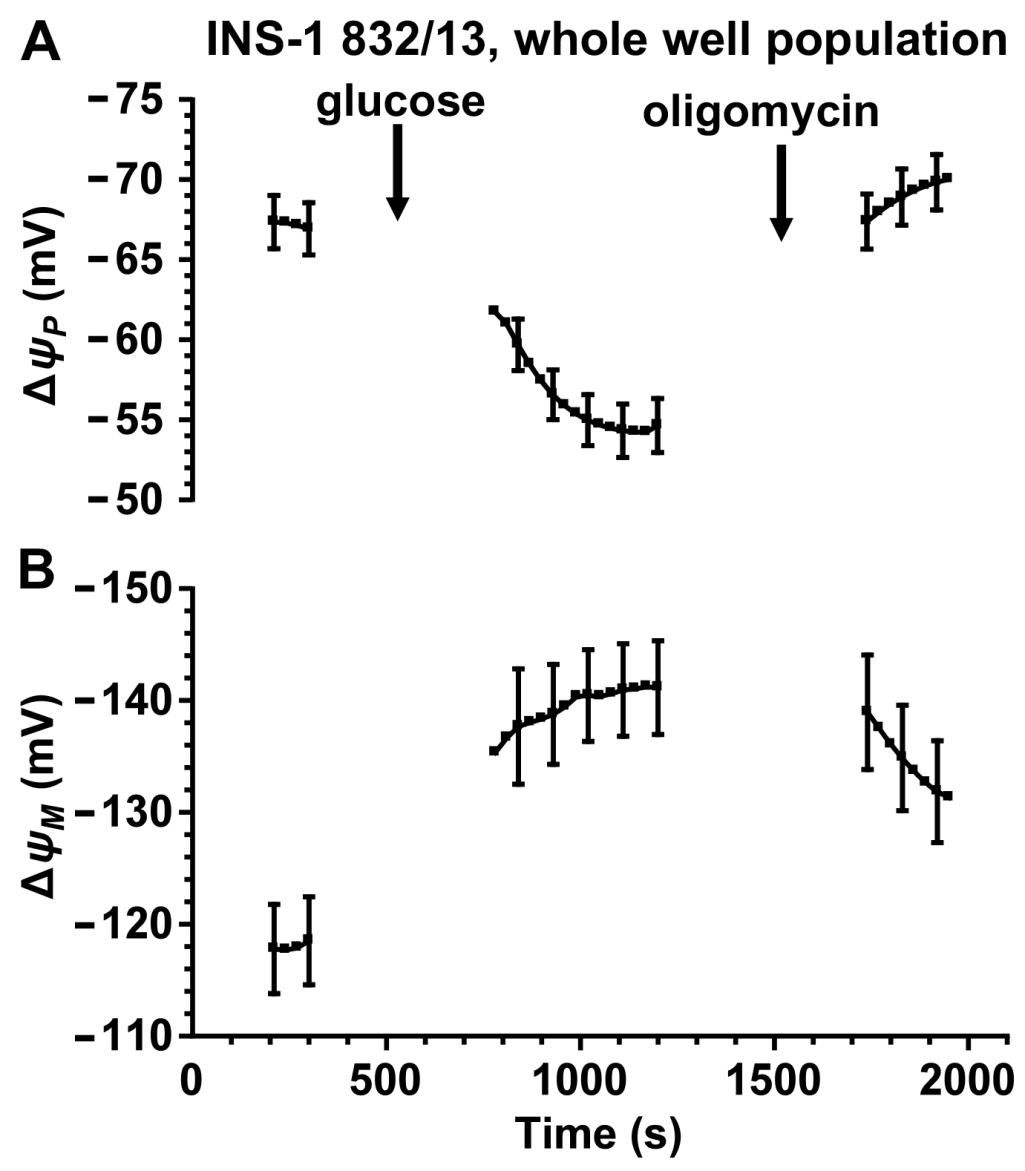
Glucose stimulation and oligomycin inhibition of INS-1 832/13 cells followed using the absolute ΔψP and ΔψM assay. (A) ΔψP and (B) ΔψM were assayed by measuring whole-well fluorescence of TMRM and PMPI in a microplate reader. Cultures were stimulated with glucose (10 mM) and treated with oligomycin (2 μg/ml) as indicated. Fluorescence traces were calibrated based on the same principles as shown in Fig 2; the time courses are shown truncated before calibration. Data are mean±SE of n = 10 independent experiments.

We demonstrate here that addition of oligomycin results in a decrease in quench-mode fluorescence of rhodamine 123 localized to the mitochondrial compartment (Fig 7B and 7F) indicating a decrease in the apparent quench limit in INS-1E and INS-1 832/13 cells at typical loading conditions of rhodamine 123 (10 μg/ml for 30 min). The mitochondrial fluorescence of rhodamine 123 was determined using the high pass filtering technique with the originally published filter [27]. This indicates that total cellular fluorescence of quench-mode rhodamine 123 cannot be used to measure the effects of oligomycin on ΔψM. However, as follows from the explanation above, rhodamine 123 fluorescence can be correctly interpreted in mitochondrion-free areas of the cell (Fig 7C). In contrast to the total cellular fluorescence, mitochondrion-free areas indicate no oligomycin-induced change in ΔψM. This is confirmed by assaying ΔψM using the non-quench mode, absolute calibrated assay. This was done using a microplate reader implementation of the technique (Gerencser et al unpublished) using the V_F_ and a_R_’ values derived above. Data from INS-1 832/13 cells using the absolute calibrated assay is consistent with the observation using rhodamine 123 over the nuclei that oligomycin does not further hyperpolarize INS-1 832/13 cell mitochondria in the presence of high (10 mM) glucose (Fig 8). Interestingly, we found similar behavior of ΔψM in non-diabetic human primary β-cells [20], and we interpreted this as the ability of high [glucose] to drive ΔψM (and therefore the ATP/ADP ratio) to the “maximum” defined in the presence of oligomycin. Strikingly, in type 2 diabetic β-cells oligomycin evoked further ΔψM hyperpolarization in high glucose [20] highlighting a deficit in the extent of glucose-evoked ΔψM hyperpolarization. Thus, in this respect, INS-1 832/13 cells had similar bioenergetic responses to the healthy human primary β-cells.

## Discussion

We have designed [21], applied [20], and simplified, improved and validated (this study) a novel technology for unbiased determination of the absolute value of ΔψM in intact cells. Here, we adapted this technique to single primary β-cells, estimated the standard error for each calibrated cell, and performed sensitivity analysis for using distribution plots of ΔψP and ΔψM in single-cells. Error estimation provided QC for single-cell data and validated the technique. Single-cell analysis of how ΔψP is linked to ΔψM indicated a variable bioenergetic activation in glucose-stimulated β-cells and operations of the canonical and possibly amplification pathways of GSIS. The significance of this novel technology is highlighted by our recent study [20], which revealed a bioenergetic signature of type 2 diabetes in human β-cells, and the validity of these data is further supported by the findings presented here.

Previous assays of ΔψM in relation to insulin secretion have been qualitative or semi-quantitative (see [33,34]). Changes in ΔψM have typically been expressed as changes of fluorescence intensity from a baseline, to normalize for sample quantity and detector settings. In earlier studies on primary β-cells and insulinoma lines [5,6,31,32], this approach did not allow for unbiased comparison, because quench-mode fluorescence changes also depend on mitochondria:cell volume fractions and quench limits that may be different between samples. Importantly, we have shown here that the quench limit of rhodamine 123 appears to change during an experimental time course critical to the evaluation of β-cell energy metabolism (Fig 7). Changes of ΔψM deduced from non-quench mode fluorescence have been previously contaminated by changes in ΔψP [10,15,35,36], a biasing factor that the absolute potentiometric technique corrects for. The combination of TMRM and PMPI and computational correction for the effects of ΔψP on TMRM accumulation has been in place before [37,38], however many subtleties of probe distribution were not accounted for and absolute potentials were not calculated (see [21]). Other techniques used for assaying ΔψM include the fluorescence probe JC-1, which is non-quantitative despite its ratiometric application, for a number of reasons [28,39], and bulk radioactive isotope distribution assays [40], which are inconvenient because of their long equilibration times, end-point nature and large specimen requirement, or specialized cases of using TMRM or TMRE in cells with large and stationary mitochondria, e.g. cardiomyocytes [41], that are not applicable to β-cells.

ΔψM is the main component of the mitochondrial proton motive force, which is proximally upstream of the ATP/ADP ratio. Precise determination of the cytoplasmic ATP/ADP ratio is currently hindered by biological factors (compartmentalization and binding [9,42,43]) and by working range and calibration issues with fluorescent ATP/ADP sensors, and this makes ΔψM a more sensitive reporter of bioenergetic forces driving GSIS than the ATP/ADP ratio. Furthermore, potentials are intensive quantities, therefore when comparing samples, normalization to cell mass is avoided, improving precision. This is a remarkable advantage compared to cell respiration, which is an extensive property. Although ΔψM is not accessible using electrophysiological recordings, most knowledge about β-cell ΔψP originates from whole-cell recordings [44]. Critical to β-cell physiology, measuring absolute values of ΔψM and ΔψP using fluorescence does not disrupt the plasma membrane or the intracellular milieu, unlike electrophysiology, which is a very significant advantage. In addition, orders of magnitude more cells can be sampled in parallel using single-cell fluorescent techniques than electrophysiological ones.

Our data (this study for rat primary β-cells and INS-1 832/13 insulinoma cells and [20] for human primary β-cells) quantify for the first time the magnitude of glucose-evoked activation of mitochondrial polarization (-34 to -45 mV; [20], Figs 4 and 8). To illustrate the impact of the magnitude of this ΔψM hyperpolarization, it could increase the maximum available rate of ATP synthesis about 100-fold [45] and the cytosolic ATP/ADP ratio to about 500-fold [46]. This strong dependence of mitochondrial energy metabolism on substrate supply is central to the operation of the canonical pathway of GSIS. Metabolic control theory [47] predicts that ΔψM is determined by the interplay of activities of supplying pathways (from glycolysis to respiratory chain) and demand pathways (ATP synthesis and cellular ATP demand). This implies that the balance between the activities of these pathways is critical for GSIS. We have proposed that a disturbance of this balance may degrade GSIS in type 2 diabetic human β-cells [20].

The value of ΔψM in non-diabetic human β-cells at low glucose (~-130 mV in humans and -120 mV in rats) is somewhat smaller than the value (~-140 mV) we found in rat neurons [21] or human stem cells [48] using the same technology, while in β-cells in high glucose it is similar (~-160 mV) to electrically activated neurons [21]. Thus ΔψM is regulated in β-cells in a similar range as in other cell types. Altogether, these results suggest that β-cell energy metabolism is not specialized in terms of capacity, but rather in its response to nutrients to support GSIS.

The glucose response by dispersed β-cells (Fig 4) was left-shifted compared to published data on intact islets [49–51], but was similar to the glucose-dependence of Ca^2+^-responses in dispersed rat β-cells [15]. A possible explanation of this is either that the physiology of dispersed β-cells differs from that of intact islets due to lack of cell to cell contacts, or that the lack of circulation in intact isolated islets poses a diffusional barrier to glucose, and requires higher external glucose concentrations to achieve the same level of stimulation.

Working with limiting amounts of human organ donor or rodent islets requires micro-scale assays. The single-cell approach maximizes the amount of data obtainable from these specimens. The cell dispersion [20], culturing and QC paradigms decrease bias from sampling islets from different parts of the pancreas or sampling cells from different parts of islets [20]. Cell viability is ensured by culturing and by single-cell QC during data analysis. In comparison, another key bioenergetic assay, respirometry, requires about tenfold more material per assay well when performed using Seahorse cell respirometry [22,52] or 150-fold more per assay for flow-through respirometry [53,54].

We exploited cell-to-cell heterogeneity to describe the relationship of ΔψM hyperpolarization and activation of ΔψP signaling in human β-cells [20], and here we showed that a very similar relationship exists in rat β-cells (Fig 6). The tight linear spread of the ΔψP– ΔψM distribution data measuring the effect of glucose (Fig 6B) suggests that the heterogeneity in ΔψP was caused by different levels of mitochondrial polarization, and all coupling factors that affect plasma membrane were linked to ΔψM uniformly for the cell population. In contrast, a cell-to-cell heterogeneous response downstream of ΔψM would lead to a poor association between ΔψP and ΔψM, rather than a tight linear spread of values. Importantly, we have demonstrated the resolution of the approach by observing the distinguishable effects of the downstream effector glibenclamide and by estimating the effects of experimental errors from various sources. This sensitivity analysis was important because it allowed us to reject the possibility that confounding factors may have imposed an artefactual relationship between ΔψM and ΔψP. Overall, measurements and modeling verified that our observations cannot be explained by factors interfering with the accuracy of the calibration, and therefore must reflect an inherent property of β-cell physiology. These data also support the possibility of substantial variations in the amounts of mitochondria per cell in individual β-cells.

Our recordings using quench-mode rhodamine 123 indicate that fluorescence data collected from whole cells or cultures can be misleading. Importantly, we recapitulated a commonly-used paradigm and it resulted internally inconsistent data (Fig 7) that was also inconsistent with the results of the absolute calibrated assay (Fig 8). Data indicated that the principle of quench–mode assaying of ΔψM does not hold in our experiments, and the fluorescence intensity of rhodamine 123 measured over the mitochondria changes during the recording. This may happen if ultrastructural changes induced by oligomycin [55] affect the quench limit, but also if mitochondria respond heterogeneously and some of the probe disappears by redistributing from completely depolarized mitochondria to charged ones, where the probe is subsequently quenched. Importantly, data obtained by quench-mode fluorescence of rhodamine 123 measured over the nucleus, which is devoid of mitochondria (Fig 7), are consistent with data from the absolute calibrated assay (Fig 8). Notably, we used the latter approach to study possible fluctuations in ΔψM in INS-1 832/13 cells in our recent study [56]. The potentiometric technique described here uses TMRM in non-quench mode, therefore changes in quench limit do not compromise the precision of the calibration.

## Conclusions

In conclusion, the work presented here has simplified the absolute potentiometric assay of ΔψM in pancreatic β-cells by replacing the multi-component ‘complete depolarization cocktail’ [21] with PFA, improved it by adding error propagation and QC, validated it by analyzing and eliminating various artefactual explanations of the observed relationships between ΔψM and ΔψP, and by showing how it is superior to commonly-used determinations using rhodamine 123. Application of the absolute calibrated ΔψM assay in β-cells could help further understanding of subtleties of GSIS by delineating relationships between the bioenergetic driving force, secondary coupling factors and insulin secretion, and aid identification of mechanisms that regulate the balance of cellular energetics.

## Abbreviations

a_R_’: activity coefficient ratio
ΔψM: mitochondrial membrane potential
ΔψP: plasma membrane potential
FCCP: carbonyl cyanide-*4*-(trifluoromethoxy)phenylhydrazone
GSIS: glucose-stimulated insulin secretion
K_ATP_: ATP-sensitive K^+^-channels
PFA: paraformaldehyde
PM: potentiometric medium
PMK: K^+^-based potentiometric medium
PMPI: ΔψP indicator
QC: quality control
SD: standard deviation
SE: standard error
TMRM: tetramethylrhodamine methyl ester
TPB: tetraphenylborate
V_F_: mitochondria:cell volume fraction
