# Correction: Measurement of the Absolute Magnitude and Time Courses of Mitochondrial Membrane Potential in Primary and Clonal Pancreatic Beta-Cells

**DOI:** 10.1371/journal.pone.0160395

**Published:** 2016-07-29

**Authors:** The PLOS ONE Staff

## Notice of Republication

This article was republished on July 19, 2016, to replace incorrect images appearing as Figs 1–8 that were introduced during preparation of the article for publication. The publisher apologizes for the errors.
